# Psychopathology dimensions and the late positive potential in adolescent and young adult females

**DOI:** 10.3758/s13415-025-01330-z

**Published:** 2025-07-29

**Authors:** Kelly A. Gair, Greg Hajcak, Brady D. Nelson

**Affiliations:** 1 Department of Psychology, Binghamton University, Binghamton, NY USA; 2 Department of Psychology, Stony Brook University, Stony Brook, NY USA; 3 School of Education and Counseling Psychology, Santa Clara University, Santa Clara, CA USA

**Keywords:** Adolescence, Late positive potential, Internalizing, Externalizing

## Abstract

Maladaptive emotional processing is central to multiple forms of psychopathology, including both internalizing and externalizing disorders. The late positive potential (LPP) is an event-related potential component that reflects attention allocation and the motivational significance of affective stimuli. Altered LPP amplitudes to emotional pictures have been observed across a range of psychopathologies, yet it remains unclear whether specific higher-order psychopathology dimensions might explain these associations. In a sample of 172 female participants aged 13 to 22 years (*M*_*age*_ = 17.85, standard deviation [*SD*] = 1.96), the present study examined the association between higher-order psychopathology dimensions and the LPP. Participants completed the Inventory of Depression and Anxiety Symptoms - Expanded Version to assess internalizing symptoms, and a parent (89.3% mothers) completed the Child Behavior Checklist to report on their child’s externalizing behaviors. Participants also completed the emotional interrupt task while electroencephalography (EEG) was recorded to measure the LPP during picture viewing. We conducted an analysis of covariance to examine the relationship between multiple psychopathology dimensions and both the overall LPP and emotion-modulated LPP. Results indicated that higher distress scores were associated with a more blunted overall LPP, while higher fear/obsessions and rule-breaking behavior scores were associated with a larger overall LPP. No psychopathology dimension was associated with the emotion-modulated LPP. The present study suggests that internalizing and externalizing subfactor dimensions are independently associated with the LPP, primarily reflecting general neural engagement with neutral, pleasant, and unpleasant emotional images.

**Supplementary Information:**

The online version contains supplementary material available at 10.3758/s13415-025-01330-z.

Adolescence is characterized by significant physical, cognitive, and emotional changes and is a critical developmental period for the emergence of both internalizing and externalizing psychopathology (Hankin et al., 2015; Kessler et al., 2005). Internalizing and externalizing disorders collectively affect a significant proportion of adolescents, with internalizing disorders, including mood and anxiety disorders, affecting approximately 20% to 30%, and externalizing disorders, such as behavior and substance use disorders, affecting approximately 10% to 20% (Merikangas et al., 2010). The incidence of these disorders often varies across developmental stages, with internalizing disorders typically emerging in late childhood to early adolescence and externalizing disorders frequently presenting in early childhood (Achenbach et al., 1991; Kessler et al., 2005). Internalizing disorders often co-occur with externalizing disorders; several have overlapping symptoms, such as attentional biases to emotional stimuli and emotional dysregulation (Beauchaine & Cicchetti, 2019; Gilliom & Shaw, 2004; Jenness et al., 2021). The frequent co-occurrence of internalizing and externalizing symptoms across development suggests they may influence one another over time and reflect shared underlying mechanisms.

Across these disorders, the developmental psychopathology framework emphasizes both homotypic continuity, where symptoms persist in a similar form over time, and heterotypic continuity, where early symptoms evolve into different forms of psychopathology (Rutter et al., 2006; Shevlin et al., 2017). For example, longitudinal studies have revealed trajectories of both pure and co-occurring internalizing and externalizing problems presenting in early childhood and continuing into adolescence (Fanti & Henrich, 2010). Over time, youth with psychiatric disorders are likely to continue experiencing the same issues or develop new ones, such as anxiety leading to depression or substance abuse; with these patterns more pronounced among girls (Costello et al., 2003). These findings underscore the importance of identifying transdiagnostic mechanisms that may contribute to the emergence and maintenance of co-occurring symptoms.

To better capture the structure of comorbid symptoms across disorders, researchers have increasingly turned to dimensional models of psychopathology, such as the Hierarchical Taxonomy of Psychopathology (HiTOP) model (Kotov et al., 2017), which emphasize both shared and distinct features of internalizing and externalizing disorders (Hankin et al., 2015; Watson, 2005). Within this framework, internalizing disorders are often divided into two primary subfactors of distress and fear (Lahey et al., 2011; Kendler et al. 2003; Slade & Watson, 2006). The distress subfactor encompasses symptoms, such as sadness, guilt, and low self-esteem, commonly associated with depression and generalized anxiety disorder, whereas the fear subfactor commonly comprises symptoms indicative of heightened arousal and reactivity to threat, characteristic of panic disorder, social phobia, and specific phobias (Watson, 2005; Lahey et al., 2011). Furthermore, externalizing disorders can be subdivided into subfactors of hyperactivity, aggressive behaviors, and delinquency/rule-breaking behaviors (including substance abuse) (Liu, 2004; Tackett, 2010). Recent research has identified specific dimensions, such as aggressive behaviors, rule-breaking, and withdrawn/depressed symptoms, that may function as bridge symptoms, contributing to the co-occurrence of internalizing and externalizing psychopathology (Liu et al., 2023). Examining these intermediate-level symptom dimensions may help clarify how overlapping symptom features contribute to broad patterns of co-occurring difficulties.

While dimensional models enhance our understanding of symptom structure, neurobiological markers may further clarify the mechanisms underlying these associations. Given their prevalence and common co-occurrence, there is a growing interest in identifying neural indicators of emotion-related processes that may help explain shared features of both internalizing and externalizing psychopathologies. Event-related potentials (ERPs), particularly the late positive potential (LPP), have emerged as promising indicators of the neural underpinnings of emotional processing (Nelson & McCleery, 2008; MacNamara et al., 2022). The LPP is a sustained positive deflection in the ERP waveform that emerges approximately 200ms after stimulus onset and reflects sustained attentional engagement and elaborative processing of emotionally salient stimuli (Hajcak et al., 2013). The LPP is typically elicited in response to viewing neutral, pleasant, and/or unpleasant images. All three categories of stimuli can evoke an LPP, and the overall LPP amplitude can serve as a measure of general attentional engagement with emotional content, irrespective of valence. Emotional stimuli tend to elicit larger LPP responses than neutral stimuli (Cuthbert et al., 2000; Bradley, 2009), particularly for pleasant or unpleasant stimuli compared to neutral stimuli (Schupp et al., 2000). Therefore, researchers often examine the emotion-modulated LPP (i.e., changes in the LPP during pleasant or unpleasant stimuli compared to neutral stimuli) as a neural indicator of emotional reactivity. Each approach to measuring the LPP has strengths and limitations: condition-level LPPs offer stronger psychometric reliability, whereas difference scores may have lower reliability but offer greater specificity for capturing emotional reactivity (Moran et al., 2013; Weinberg & Hajcak, 2011). Importantly, the LPP has demonstrated robust sensitivity across developmental stages, including early childhood (Hua et al., 2014), adolescence (Kujawa et al., 2013), and adulthood (Moran et al., 2013).

Across adolescence and adulthood, internalizing psychopathology has been consistently associated with alterations in the emotion-modulated LPP, which reflects neural responses to emotional versus neutral stimuli. Specifically, individuals with depression or generalized anxiety disorder often exhibit a blunted LPP to unpleasant stimuli relative to neutral stimuli, suggesting reduced emotional reactivity (Foti et al., 2010; Weinberg et al., 2016). These findings are frequently interpreted as evidence of diminished neural engagement with negative emotional content in distress-based disorders. In contrast, studies of fear-based disorders using similar difference score methods have found heightened LPP responses to unpleasant stimuli. For instance, individuals with panic disorder (Pauli et al., 1997), social phobia (Moser et al., 2008), and specific phobias (Michalowski et al., 2009; Miltner et al., 2005) demonstrated enhanced LPP amplitudes to threat-relevant stimuli compared to neutral stimuli. This pattern reflects heightened neural sensitivity to negative emotional information in fear-related conditions, in contrast to the dampened reactivity observed in distress-based disorders.

Other research has taken a condition-level approach, examining LPP responses to specific emotional conditions without computing difference scores. These studies provide a complementary perspective by focusing on neural responses to particular types of emotional content. For instance, longitudinal research has found that blunted LPP responses to unpleasant stimuli at baseline predicted the future onset of depressive disorder in adolescent girls (Michelini et al., 2021). In addition, some evidence suggests that this blunted response may extend beyond negative stimuli. For instance, individuals with depression also show reduced LPP amplitudes to pleasant stimuli, suggesting a broader disengagement from emotionally salient content regardless of valence (Bylsma, 2021). Notably, this pattern of altered neural response to emotional stimuli is not limited to internalizing disorders. Emerging evidence suggests that similar reductions in LPP amplitude may also occur in externalizing populations. For example, a diminished LPP response to pleasant stimuli, such as happy faces, has been associated with increased rule-breaking behaviors and social difficulties in anxious youth (Bunford et al., 2017). Likewise, other studies have reported a diminished LPP to unpleasant stimuli, such as negative emotional words or threatening images, in individuals exhibiting psychopathic traits and binge drinking behaviors (Sadeh & Verona, 2012; Medina et al., 2016; Connell et al., 2015), indicating that blunted emotional reactivity may also play a role in externalizing behaviors. However, research examining higher-order externalizing symptom dimensions has found no significant relation with the LPP (Rozalski & Benning, 2019). Thus, while there is evidence suggesting a connection between the LPP and externalizing psychopathologies, the precise nature of this relationship remains unclear. These condition-level studies, though not directly contrasting emotional and neutral stimuli, offer important insight into how individuals with diverse symptom profiles engage with specific types of emotional content.

In parallel, a smaller body of research has examined internalizing symptoms in relation to overall LPP amplitude, independent of stimulus valence. These studies offer a more global perspective on neural engagement, suggesting that distress-based disorders are associated with a generalized reduction in LPP amplitude across all stimulus types. For instance, Nelson et al. (2015) found that adolescent girls with higher depressive symptoms exhibited reduced LPP amplitudes regardless of emotional content. Granros et al. (2022) further supported this distinction by demonstrating that distress/misery disorders were associated with a blunted overall LPP, while fear-based disorders were associated with an enhanced overall LPP response, even in the absence of stimulus-specific contrasts. Additionally, Fedorenko et al. (2023) found that among trauma-exposed undergraduates, depressive symptoms were inversely related to the LPP response to neutral images, with no associations found for positive or negative stimuli. This suggests that blunted LPP activity may extend even to non-affective or ambiguous content in the context of elevated depressive symptoms.

The complex relation between the LPP and different disorders may be better understood through higher-order models of psychopathology that cut across traditional diagnostic categories. The present study is guided by the HiTOP framework, which posits that psychopathology can be understood along shared dimensions, including internalizing and externalizing spectra (Kotov et al., 2017). This approach allows for a more refined understanding of how specific symptom clusters, such as distress, fear, aggressive behavior, and rule-breaking behavior, may relate to underlying patterns of neural activity. By leveraging this dimensional model, the current study seeks to clarify how these transdiagnostic dimensions map onto neural responses to emotional stimuli, as indexed by the LPP.

The present study extends prior research by investigating, in a sample of 172 females aged 13 to 22 years, the association between higher-order internalizing and externalizing psychopathology subfactor dimensions and the LPP. Specifically, this study explored whether distinct subfactors, namely distress, fear, positive mood, aggressive behavior, and rule-breaking behavior, are differentially associated with the overall LPP (reflecting general attentional engagement) or emotion-modulated LPP (reflecting emotional reactivity) in adolescence and young adulthood. Based on existing literature, we hypothesized distinct associations between these subfactors and the LPP, specifically that higher distress subfactor scores will relate to a smaller overall LPP and emotion-modulated LPP to pleasant and unpleasant images, and higher fear subfactor scores will relate to a larger overall LPP and emotion-modulated LPP to unpleasant images. Existing literature has found mixed relations between the LPP and externalizing problems; therefore, we did not have specific directional hypotheses regarding the LPP and aggressive or rule-breaking behaviors. Additionally, positive mood was included as a distinct dimension to explore its potential contribution to emotional processing, and we did not have specific a priori hypotheses for this analysis. This investigation aims to extend our understanding of the distinct neural correlates of both internalizing and externalizing psychopathology, offering a more nuanced view of how shared mechanisms, specifically emotional processing measured by the LPP, vary across these higher-order factors.

## Method

### Participants

The sample consisted of 193 females aged 13 to 22 years and a biological parent (89.3% mothers) who participated in the third wave of a longitudinal study exploring trajectories of reward sensitivity and depression across adolescence and young adulthood. The overarching study focused on adolescent girls as they are the demographic group at greater risk for first-onset depression. The third wave was selected for analysis due to the introduction of the Inventory of Depression and Anxiety Symptoms - Expanded Version (IDAS-II; Watson et al., 2012), which allowed for the examination of multiple internalizing symptom dimensions across the developmental period. While the study predominantly focused on adolescence, the inclusion of young adults in these analyses helped capture developmental transitions extending beyond adolescence. Participants were excluded from data analysis due to poor performance (<75% correct responses) on the emotional interrupt task (*n* = 5) or missing IDAS-II (*n* = 5) or CBCL (*n* = 11) scores, resulting in a final sample of 172 (*M*_*age*_ = 17.85, standard deviation [*SD*] = 1.96). The racial distribution of the current sample was 83.4% White, 6.2% Black, 0.5% Native American, and 6.2% more than one race, and the ethnic distribution was 9.8% Hispanic. The education level of the participants was such that 37.4% completed partial college or graduated college, 60.2% completed partial high school or graduated high school, and 2.3% completed junior high or middle school. Participants were recruited via commercial mailing lists, fliers, and online postings within a 30-mile radius of Stony Brook, New York, and enrolled into the study if the child was within age range without any developmental or medical disabilities and had an English-speaking biological parent who was also willing to participate in the study. All participants provided written assent or informed consent and were financially compensated for their participation, with parental/guardian signatures required for minors to accept payment. All tasks and procedures were approved by Stony Brook University’s Institutional Review Board.

### Measures

#### Inventory of depression and anxiety symptoms - expanded version (IDAS-II)

The IDAS-II (Watson et al., 2012) is a 99-item self-report questionnaire that measures 18 factor-analytically derived symptom components of depression, anxiety, and bipolar disorders. Participants rate symptoms over the past two weeks using a 5-point Likert scale ranging from “*not at all*” (1) to “*extremely*” (5), with higher scores indicating greater severity. The present study examined the distress, fear/obsessions, and positive mood subfactor composites delineated from this measure using factor loadings set forth by Watson et al. (2012), which were further supported by normative data from a large, nationally representative sample in Nelson et al. (2018), enhancing the measure’s applicability across demographic groups. In the current sample, all IDAS-II subscales demonstrated acceptable internal consistency, ranging from α = .71 (euphoria) to α = .90 (dysphoria, cleanliness).

#### Child behavior checklist - parent report form (CBCL)

The CBCL (Achenbach, 2001) is a parent-report measure of 6- to 18-year-old youth externalizing and internalizing problems. Although the CBCL is traditionally used for assessing children and adolescents, its use in the present study with young adults up to age 22 is justified to maintain consistency with earlier waves of this longitudinal study, ensuring comparability over time. Parents, primarily mothers, rated how descriptive each item was of their child’s usual behavior now or within the past 6 months on a 3-point scale (0 = *not true;* 1 = *somewhat or sometimes true*; 2 = *very true or often true*) (Achenbach, 2001). The present study focused on raw scores from the externalizing rule-breaking behavior (17 items) and aggressive behaviors (18 items) scales. In the current sample, the CBCL subscales demonstrated acceptable internal consistency (aggressive behavior α = .71, rule-breaking behavior α = .69).

The multi-informant approach of using the IDAS-II to assess self-reported internalizing symptoms and the CBCL to assess parent-reported externalizing symptoms was chosen to capitalize on the strengths of each informant type. Self-report is ideal for capturing the subjective experience of internalizing symptoms, such as depression and anxiety, which may not be easily observable by others (De Los Reyes et al., 2015). In contrast, externalizing behaviors, such as rule-breaking and aggression, are more visible to others and are often better reported by parents who have long-term observational insights into their child’s behaviors (Achenbach et al., 1987). This multi-informant strategy serves to enhance the validity of our assessments by incorporating complementary perspectives on the participants’ behavioral and emotional functioning.

### Procedure

After assent and informed consent were obtained, participants completed a modified version of the emotional interrupt task (Mitchell et al. 2006; Weinberg & Hajcak 2011), administered using Presentation software (Neurobehavioral Systems, Inc.). The third wave assessment included diagnostic interviews, self- and parent-reported questionnaires, EEG recordings, and fMRI scans. Data for the present study were obtained from one EEG task, one self-report questionnaire, and one parent-reported questionnaire. Specific to the current study, participants were asked to respond to a left- or right- pointing arrow that was displayed in between the presentation of an emotional picture. Each trial consisted of a fixation point (800ms), followed by a neutral, pleasant, or unpleasant picture (1,000 ms), then followed by either a left- (<) or right- (>) pointing arrow (i.e., the target; 150 ms), followed by the same picture that preceded the target (400ms). Participants were instructed to view the images as they were presented and press the left or right computer mouse button to indicate the direction of the arrow on the screen. Between trials there was a blank screen ranging from 1,500 to 2,000 ms. The task included 120 trials (40 neutral, 40 pleasant, 40 unpleasant) presented to the subject in a random order. Stimuli for the task included 60 pictures deemed developmentally appropriate for children from the International Affective Picture System (IAPS; Lang et al., 2008). Among these images, 20 displayed pleasant images (e.g., smiling children, cute animals), 20 neutral images (e.g., outdoor benches, household items), and 20 unpleasant images (e.g., angry faces, aggressive animals).^1^ Arousal and valence ratings confirmed expected differences between pleasant (valence: *M* = 7.51, *SD* = 1.5; arousal: *M* = 5.03, *SD* = 2.28), neutral (valence: *M* = 5.27, *SD* = 1.27; arousal: *M* = 2.99, *SD* = 1.91), and unpleasant images (valence: *M* = 3.09, *SD* = 1.71; arousal: *M* = 6.12, *SD* = 2.11). See supplemental materials for comparison of normative arousal and valence ratings of the images. Although we aimed to select images that were both developmentally appropriate and distinct in emotional valence, unpleasant images were significantly more arousing than pleasant images, as we were unable to utilize the higher-arousal pleasant images that were not appropriate for youth (i.e., erotica images).

### Electroencephalography recording and data processing

Continuous electroencephalogram (EEG) was recorded using a 34-channel ActiveTwo BioSemi system (BioSemi, Amsterdam, Netherlands) according to the 10/20 system with two additional electrodes placed on the left and right mastoids. Electrooculogram (EOG) activity from eye movements and eye blinks were recorded using four facial electrodes: two placed approximately 1 cm outside of the left and right eyes, and two placed approximately 1 cm above and below the right eye. The EEG data was digitized at a sampling rate of 1024 Hz. EEG data were analyzed using BrainVision Analyzer 2.2 (Brain Products, Gilching, Germany). We used a semiautomatic processing procedure to detect and reject data artifacts. Intervals with a voltage step of more than 50 μV between sample points, a voltage difference of 300 μV within a trial, and a maximum voltage difference of less than 0.5 μV within 100-ms intervals were rejected from individual channels in each trial. We then visually inspected the data to detect and reject remaining artifacts.

The LPP was segmented between 300 and 1000 ms after picture onset. This time window was selected based on both visual inspection of the grand average waveform and alignment with prior literature identifying this window as capturing sustained LPP activity in affective tasks. Mean amplitude was calculated following stimulus onset, at a pooling of electrodes Oz, O1, and O2 separately for pleasant, neutral, and unpleasant images. We only used the trials for which the behavioral response to the arrow was correct, to increase the likelihood participants were paying attention and viewing the image during each trial. The average number of trials (out of 40) used for each condition were 38.40 (*SD* = 2.54) for neutral, 38.58 (*SD* = 2.13) for pleasant, and 38.10 (*SD* = 2.88) for unpleasant. The odd-even split-half reliability, corrected using the Spearman-Brown formula, for the LPP in each condition was .88 for neutral, .87 for pleasant, and .88 for unpleasant.

### Data analysis

The bivariate associations between the psychopathology measures and the LPPs were analyzed using Pearson correlations. The comparison of the LPP amplitude and behavioral performance (accuracy in response to arrow) across different valence stimuli were each analyzed using a repeated measures analysis of variance (ANOVA). The relationship between the LPP and psychopathology dimensions was analyzed using a mixed-measures analysis of covariance (ANCOVA) with valence (neutral, pleasant, and unpleasant) as the within-subject factor, and psychopathology dimensions (distress, fear/obsession, positive mood, aggressive behavior, rule-breaking behavior) as the covariates. Main effects of psychopathology dimensions indicated relationships with the overall LPP and were followed up with a linear regression with all psychopathology dimensions as independent variables and the average LPP across all three conditions (neutral, pleasant, and unpleasant) as the dependent variable. Significant Psychopathology X Valence interactions indicated relationships with the emotion-modulated LPP and were followed-up by examining the association between each psychopathology symptom dimension residual (controlling for the other symptom dimensions) and LPP enhancement to pleasant (i.e., pleasant-neutral) and unpleasant (i.e., unpleasant-neutral) stimuli. This analytical approach allowed us to examine both general emotional engagement (via main effects) and emotional reactivity (via interactions with valence). Identical ANCOVAs were conducted with the psychopathology measures and behavioral performance (accuracy in response to arrow). All ANCOVA analyses were conducted in IBM SPSS Statistics, version 29.0 (USA).

## Results

Table 1 displays Pearson’s correlations and descriptive statistics for the IDAS-II distress, fear/obsessions, and positive mood scales, the CBCL aggressive behavior and rule-breaking behavior scales, and the LPP to neutral, pleasant, and unpleasant images. Results indicated positive correlations between multiple higher-order psychopathology dimensions. Specifically, distress factor scores were positively correlated with fear/obsession factor scores, indicating a strong association between these two internalizing dimensions. Similarly, aggressive behavior factor scores were positively correlated with rule-breaking factor scores, demonstrating a strong association between the two externalizing dimensions as well. Additionally, distress factor scores were positively associated with both aggressive and rule-breaking behavior factor scores, suggesting that higher levels of distress are associated with greater externalizing behaviors. Positive mood was not associated with any other dimensions.

**Table 1:** Descriptive statistics and bivariate correlations between psychopathology measures and LPP

	1	2	3	4	5	6	7	8
1. IDAS-II Distress	-							
2. IDAS-II Fear/Obsessions	**0.73**	-						
3. IDAS-II Positive Mood	−0.14	**0.22**	-					
4. CBCL Aggressive Behavior	**0.34**	0.13	−0.13	-				
5. CBCL Rule-Breaking Behavior	**0.34**	**0.16**	−0.13	**0.73**	-			
6. LPP to Neutral Stimuli	0.01	0.13	−0.02	0.10	0.15	-		
7. LPP to Pleasant Stimuli	0.04	0.10	−0.09	0.07	**0.17**	**0.83**	-	
8. LPP to Unpleasant Stimuli	0.08	0.13	−0.05	0.08	**0.16**	**0.80**	**0.82**	-
Mean	62.31	29.13	23.36	2.60	1.31	8.30	9.01	10.51
Standard Deviation	21.45	9.09	6.39	3.48	1.97	5.74	5.73	5.95

*Note*. All bolded correlations are statistically significant at *p* < .05

CBCL = Child Behavior Checklist; IDAS-II = Inventory of Depression and Anxiety

Symptoms - Expanded Version; LPP = late positive potential

**Fig. 1:**
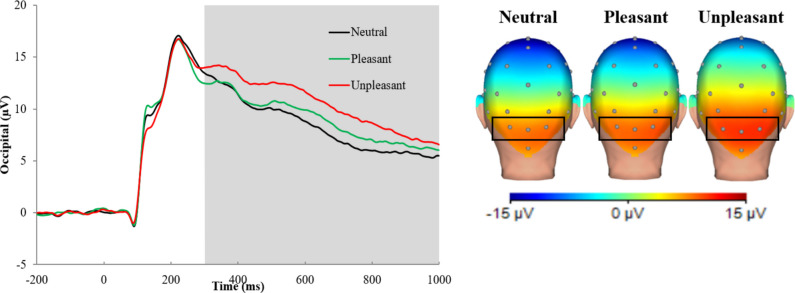
Event-related potential waveforms and scalp distributions for the emotion interrupt task. *Note*. The shaded region shows where the LPP was quantified (between 300–1,000 ms following feedback). Scalp distributions from left to right: neutral, pleasant, and unpleasant conditions. Electrodes of interest (O1, Oz, O2) are marked within the black box on the scalp maps

Figure 1 displays the LPP waveform and scalp topographies elicited by neutral, pleasant, and unpleasant stimuli. Across all participants the LPP differed across conditions, *F*(2, 342) = 35.87, *p* < .001, η_p_^2^ = .17. Specifically, the LPP was larger to unpleasant images compared to neutral images, *F*(1, 171) = 61.49, *p* < .001, η_p_^2^ = .26, and compared to pleasant images, *F*(1, 171) = 32.08, *p* < .001, η_p_^2^ = .16. Furthermore, the LPP response to pleasant images was significantly larger compared to neutral images, *F*(1, 171) = 7.93, *p* = .005, η_p_^2^ = .04. In the examination of behavioral performance, there was no main effect of condition, suggesting that accuracy of response to the arrows did not differ across the different emotional stimuli.

As shown in Table 2, the ANCOVA indicated significant main effects of IDAS-II distress, fear/obsessions, and CBCL rule-breaking behavior when averaging the LPP across all conditions. Follow-up analyses indicated that greater distress factor scores were associated with a smaller overall LPP, β = −.30, *t* = −2.26, *p* = .025, 95% confidence interval [CI]: −.14, −.01. In contrast, greater fear/obsessions, β = .35, *t* = 2.75, *p* = .007, 95% CI: .06, .36, and rule-breaking behavior, β = .23, *t* = 2.06, *p* = .041, 95% CI: .03, 1.23, scores were associated with a larger overall LPP. All three results remained statistically significant after using the Benjamini-Hochberg procedure to control for false discovery rate (Benjamini & Hochberg, 1995). There were no significant interactions between psychopathology dimensions and valence. See supplementary materials for analyses involving each individual psychopathology dimension and measure of the LPP.

**Table 2:** ANCOVA results for psychopathology dimensions and the LPP

Subfactor	*F*	*p*	*η*_*p*_^*2*^
IDAS-II Distress	5.11	**.025**	0.03
IDAS-II Fear/Obsessions	7.59	**.007**	0.04
IDAS-II Positive Mood	3.24	.074	0.02
CBCL Aggressive Behavior	0.15	.701	<.01
CBCL Rule-Breaking Behavior	4.24	**.041**	0.03
IDAS-II Distress x Valence	2.12	.122	0.01
IDAS-II Fear/Obsessions x Valence	1.11	.331	<.01
IDAS-II Positive Mood x Valence	0.80	.452	<.01
CBCL Aggressive Behavior X Valence	1.47	.233	<.01
CBCL Rule-Breaking Behavior X Valence	0.70	.496	<.01

*Note*. All bolded p-values are statistically significant at *p* < .05

IDAS-II = Inventory of Depression and Anxiety Symptoms - Expanded Version; CBCL = Child Behavior Checklist

## Discussion

In a sample of 172 females aged 13 to 22 years, the present study examined the association between internalizing and externalizing dimensions and the LPP. The primary focus was on the distinct associations between specific subfactor dimensions, including distress, fear/obsessions, positive mood, aggressive behavior, rule-breaking behavior, and the LPP in response to emotional stimuli. The subfactors were selected for their theoretical relevance to hierarchical models of psychopathology. Specifically, the HiTOP framework emphasizes the dimensional and hierarchical organization of symptoms, suggesting that these subfactors may differentially relate to neural mechanisms associated with emotional processing. Our findings support this perspective by showing that the overall LPP, reflecting general attentional engagement, relates meaningfully to the subfactor level of psychopathology. This highlights the utility of dimensional models in elucidating the neural correlates of mental health difficulties and suggests that future research may benefit from focusing on transdiagnostic symptom dimensions rather than categorical diagnoses.

Our specific hypotheses posited that distress, characterized by symptoms commonly associated with depression and anxiety, would relate to a smaller LPP, reflecting reduced neural engagement with emotional stimuli. Conversely, we anticipated that fear/obsessions would be associated with a larger LPP, indicative of heightened sensitivity to emotional stimuli. Consistent with these predictions, higher distress scores were associated with a smaller overall LPP response, while greater fear/obsessions and rule-breaking behavior scores were both associated with a larger overall LPP response. These findings reveal opposing patterns of neural engagement and contribute to a more nuanced understanding of how different psychopathology dimensions influence attention to emotional stimuli.

The observed association between higher distress scores and a smaller overall LPP aligns with prior research linking distress-related disorders to blunted emotional responses (Granros et al., 2022; Nelson et al., 2015). The present study reaffirms these findings, illustrating that greater distress subfactor scores are associated with a smaller overall LPP and greater fear subfactor scores are associated with a larger overall LPP. This reduced neural engagement could contribute to the anhedonia and emotional numbness commonly observed in distress-related disorders. Conversely, the larger overall LPP associated with greater fear scores reflects the heightened emotional reactivity typical of panic and phobic disorders (MacNamara et al., 2011; Nelson & McCleery, 2008). This heightened emotional engagement may be a characteristic feature of fear-related disorders, possibly reflecting hypervigilance and heightened arousal in response to perceived threats.

Although the LPP is often conceptualized as an index of emotional reactivity, the literature reflects diverse analytic strategies. Specifically, some studies have used difference scores (emotional minus neutral) to isolate emotional modulation (Foti et al., 2010; Wienberg & Hajcak, 2011). Other studies have focused on responses to specific emotional conditions (neutral, pleasant, unpleasant), analyzing each stimulus type separately (Michelini et al., 2021; Bylsma, 2021). Whereas others have examined the overall LPP amplitude, averaging across all conditions to examine general emotional engagement (Nelson et. al., 2015; Granros et al., 2022). In our study, we tested both emotion-modulated and condition-specific effects through a mixed-measures ANCOVA with valence as a within-subjects factor and psychopathology dimensions as continuous covariates. Results indicated main effects of psychopathology dimensions but no interactions with valence, suggesting the psychopathology measures were associated with the overall LPP but not the emotion-modulated LPP. This pattern of results is consistent with the two previous investigations that examined internalizing dimensions and the LPP (Nelson et al., 2015; Granros et al., 2022). Consistent with these findings, prior work has demonstrated reduced LPP responses to both neutral and pleasant images among individuals with depression (Klawohn et al., 2021), highlighting blunted engagement with broadly salient stimuli rather than specific emotional deficits. However, other studies have reported that emotional reactivity is most apparent when examining condition-specific LPP responses, particularly to emotional (rather than neutral stimuli). For instance, Hill et al. (2019) found attenuated LPP responses to both pleasant and unpleasant, and not neutral, stimuli in relation to depressive symptoms, Similarly, Moretta and Massarotti Benvenuti (2023) found that individuals with familial risk for depression showed blunted LPPs to pleasant and unpleasant stimuli but not to neutral images. These findings suggest that condition-specific analyses may yield effects not captured by an overall scoring approach. Taken together, the current results contribute to ongoing research that attempts to understand how to best conceptualize and score the LPP when examining its association with psychopathology.

While the LPP was largest for unpleasant stimuli compared to pleasant and neutral stimuli, this pattern likely reflects differences in arousal rather than valence. Normative IAPS ratings confirmed that the unpleasant images used in this study were significantly more arousing than the pleasant images. These condition differences are consistent with prior research indicating that the LPP is particularly sensitive to arousal (Hajcak et al., 2012; Schupp et al., 2000). Although stimulus arousal differences likely drove LPP condition effects, emotional valence remains conceptually relevant in interpreting neural engagement with affective stimuli (Weinberg & Hajcak, 2010). One potential source of variability in the literature may be attributed to variations in stimuli characteristics and selection. While the present study and the reviewed studies utilized pictures from the IAPS library, our pleasant stimuli (e.g., smiling children and cute animals) were selected to be developmentally appropriate for children and thus featured lower normative arousal ratings compared to the unpleasant images. This imbalance likely reflects a more limited availability of high-arousal, child-appropriate pleasant images in the IAPS database. Future studies should better match stimuli across valence categories on arousal and consider collecting subjective arousal and valence ratings to improve precision in interpreting LPP condition effects.

One of the major strengths of the present study was the examination of both internalizing and externalizing psychopathology. While some studies have reported associations between externalizing behaviors and deviations in neural reactivity to emotional stimuli (Bunford et al., 2017; Sadeh & Verona, 2012; Medina et al., 2016), others have looked at an overarching externalizing dimension and found no associations (Rozalski & Benning, 2019). Moreover, a recent meta-analysis found that substance users demonstrate a larger LPP to alcohol and drug cues compared to controls (Webber et al., 2022). Additional research employing other psychophysiological methodologies has found associations with externalizing behavior. For example, adults who met criteria for externalizing disorders, including conduct disorder or antisocial behavior, were found to display reduced electrodermal and electromyographic activity to emotional stimuli (Herpertz et al., 2001). Thus, these findings underscore the importance of examining externalizing symptoms in relation to neural measures, particularly given the mixed evidence to date. Building on this, the present study found that higher rule-breaking behavior scores were associated with a larger LPP response. One possible explanation for this result is that adolescents who are engaging in rule breaking behaviors may exhibit increased neural sensitivity to emotionally salient stimuli, which could be related to the heightened impulsivity and emotional dysregulation characteristic of these behaviors. We did not find an association between aggressive behaviors and the LPP, suggesting that the neural processing of emotional stimuli may not be related to more antagonistic behaviors in the same way it is to more disinhibited behaviors. This finding highlights the heterogeneity within the externalizing spectrum and the need for further research to disentangle the specific neural mechanisms underlying different lower-order dimensions of externalizing psychopathology. By demonstrating associations between both internalizing and externalizing psychopathology with changes in neural reactivity, the present study highlights the need for a more comprehensive assessment in examining the underlying mechanisms of psychopathology.

While the present study provides valuable insights into the relation between psychopathology dimensions and the LPP in adolescence, there are a few important limitations to acknowledge. The cross-sectional nature of the study limits our ability to infer causality or temporal dynamics of the observed associations. Additionally, the sample consisted of primarily non-Hispanic white females, which may limit the generalizability of the findings to more diverse and male populations. Future research should explore whether this association extends to other populations, including males, and other more racially and ethnically diverse populations. Another limitation is that we did not collect subjective ratings of valence and arousal of emotional stimuli. Subjective ratings are critical because individual differences in emotional perception can influence neural responses, including the LPP. For instance, two individuals may rate the same image differently in terms of its emotional intensity, leading to variability in neural reactivity that is not captured by standardized stimulus sets alone. Without subjective ratings, it remains unclear whether the observed LPP associations reflect differences in actual stimulus-driven neural reactivity or differences in how participants internally appraise the stimuli. Future research should incorporate valence and arousal ratings to determine whether psychopathology-related reductions in LPP amplitude are due to altered neural processing of affective content or differences in the subjective experience of emotions. This would provide a more precise characterization of how psychopathology dimensions influence emotional engagement and neural processing. Lastly, while parents are often thought to be better reporters of externalizing symptoms due to their ability to observe behavior across various contexts, relying solely on parent-report of externalizing behaviors in our study may limit the understanding of adolescents’ subjective experiences and perceptions. Future research may address this limitation by incorporating both self- and parent-report measures of internalizing and externalizing symptomatology to capture a more comprehensive assessment of psychopathology.

Adolescents who exhibit differences in the neural processing of emotionally salient stimuli may be more susceptible to developing a range of psychopathology symptoms across the internalizing and externalizing spectra. This notion is consistent with theoretical models proposing transdiagnostic processes underlying psychopathology, such as the Research Domain Criteria, which emphasize shared neurobiological and behavioral mechanisms that cut across traditional diagnostic boundaries (Cuthbert, 2014). This study highlights the importance of considering dimensional models of psychopathology in understanding the neural correlates of emotional processing in adolescents and young adult females. By identifying specific patterns of neural responses associated with distinct psychopathology dimensions, our findings offer valuable insights that may inform more targeted assessment and intervention strategies aimed at promoting mental health and well-being in this population.

## Supplementary Information

Below is the link to the electronic supplementary material.

Supplementary file1 (DOCX 22 KB)

## Data Availability

Research data has been made publicly available: https://osf.io/5kjvf/?view_only=4b7dad079d4c4fc7bc7d6c92914d292e

## Footnotes

1 Image numbers from IAPS database: pleasant (1463, 1710, 1750, 1811, 2070, 2091, 2092, 2224, 2340, 2345, 2347, 7325, 7330, 7400, 8031, 8200, 8370, 8461, 8496, 8497), neutral (2514, 2580, 5390, 5395, 5500, 5731, 5740, 5900, 7000, 7002, 7009, 7010, 7026, 7038, 7039, 7090, 7100, 7130, 7175, 7190), and unpleasant (1050, 1052, 1200, 1205, 1300, 1304, 1930, 2458, 2691, 2703, 2800, 2811, 2900, 3022, 6190, 6213, 6231, 6510, 6571, 9600).
