# Supplementary material for: Psychopathology dimensions and the late positive potential in adolescent and young adult females
Source: Cogn Affect Behav Neurosci. 2025 Jul 29;26(1):319–29. doi: 10.3758/s13415-025-01330-z (PMC13424224; doi:10.3758/s13415-025-01330-z)
Supplement: Supplementary file 1 — Supplementary file1 (DOCX 22 KB) [file 13415_2025_1330_MOESM1_ESM.docx]

**Supplemental Materials**

**Image Valence and Arousal Ratings**

We analyzed ratings from the International Affective Picture System (IAPS) database to test whether the images (20 neutral, 20 pleasant, 20 unpleasant) differed on normative valence and arousal levels. A one-way analysis of variance (ANOVA) was conducted separately for arousal and valence ratings to ensure the categories differed significantly in their emotional content. For arousal, there was a main effect, *F*(2, 60) = 121.35, *p* < .001, η^2^ = .80, such that neutral images (*M* = 2.99, *SD* = 0.66) were less arousing than both pleasant images (*M* = 5.03, *SD* = 0.75) (*p* < .001) and unpleasant images (*M* = 6.12, *SD* = 0.56) (*p* < .001), and unpleasant images were more arousing compared to pleasant images (*p* < .001). For valence, there was also a main effect, *F*(2, 60) = 338.23, *p* < .001, η^2^= .92, such that pleasant images (*M* = 7.51, *SD* = 0.50) were more pleasant than neutral images (*M* = 5.27, *SD* = 0.34) (*p* <.001) and unpleasant images (*M* = 3.09, *SD* = 0.74) (*p* < .001), and neutral images were more pleasant than unpleasant images (*p* < .001).

**Alternative Late Positive Potential (LPP) Analyses**

The present study employed an omnibus model to examine the association between psychopathology and the LPP. Researchers have used different approaches toward quantifying the LPP, including examining each condition (neutral, pleasant, and unpleasant) alone as well as difference scores for each emotional condition (pleasant-neutral, unpleasant-neutral). We conducted additional linear regression analyses using these approaches toward quantifying the LPP to make the results relevant to the broader LPP literature. See Table 1 for the results of these analyses. Briefly, smaller LPP amplitudes to neutral and pleasant images, but not unpleasant images, were associated with greater distress, and larger LPP amplitudes to neutral, pleasant, and unpleasant images were associated with greater fear/obsessions. Smaller LPP amplitudes to pleasant images were also associated with greater positive mood. Finally, greater LPP amplitudes to pleasant images were associated with greater rule-breaking behaviors. Regression analyses for difference scores did not reveal significant associations with psychopathology dimensions. There was a trend-level association between higher distress and greater unpleasant-minus-neutral LPP difference scores (*p* = .053).

**Table 1.** Linear regression results for LPP raw scores and difference scores

|  | Neutral | | Pleasant | | Unpleasant | | Pleasant-Neutral | | Unpleasant-Neutral | |
| --- | --- | --- | --- | --- | --- | --- | --- | --- | --- | --- |
| Subfactor | *β* | *p* | *β* | *p* | *β* | *p* | *β* | *p* | *β* | *p* |
| IDAS-II Distress | -.36 | .006 | -.29 | .030 | -.19 | .159 | .13 | .320 | .26 | .053 |
| IDAS-II Fear/Obsessions | .39 | .002 | .33 | .011 | .27 | .040 | -.11 | .379 | -.18 | .162 |
| IDAS-II Positive Mood | -.13 | .121 | -.19 | .032 | -.12 | .181 | -.09 | .311 | .02 | .822 |
| CBCL Aggressive Behavior | .02 | .834 | -.09 | .396 | -.05 | .664 | -.20 | .073 | -.11 | .315 |
| CBCL Rule-Breaking Behavior | .18 | .107 | .26 | .019 | .20 | .074 | .14 | .215 | .05 | .689 |

*Note*. Each column represents a separate linear regression in which the psychopathology subfactors were included as independent variables and the LPP to a specific condition was included as the dependent variable. CBCL = Child Behavior Checklist; IDAS-II = Inventory of Depression and Anxiety Symptoms – Expanded Version; LPP = late positive potential.
